# An Investigation of the Variations in Complete Mitochondrial Genomes of *Lingula anatina* in the Western Pacific Region

**DOI:** 10.3390/biology10050367

**Published:** 2021-04-25

**Authors:** Mustafa Zafer Karagozlu, Thinh Dinh Do, Jung-Il Kim, Tae-June Choi, Seong-Geun Kim, Chang-Bae Kim

**Affiliations:** 1 Department of Biotechnology, Sangmyung University, Seoul 03016, Korea; zaferka@gmail.com (M.Z.K.); deepblue.th@gmail.com (T.D.D.); kim2429@gmail.com (J.-I.K.); ctj2343@naver.com (T.-J.C.); sheller119@naver.com (S.-G.K.); 2 3Billion Inc., Seoul 06193, Korea; 3 Institute of Marine Environment and Resources, Vietnam Academy of Science and Technology, Haiphong 04000, Vietnam

**Keywords:** brachiopod, *Lingula anatina*, complete mitochondrial genome, gene arrangement, cryptic species, molecular evolution

## Abstract

**Simple Summary:**

*Lingula anatina* has been attracted researchers because its morphological characteristics show limited changes compared to the ancestor. Even though *L. anatina* is a common brachiopod in the western Pacific region, a few studies were performed to investigate genetic variations of this species. To understand the mitogenomic diversity of *L. anatina* in the region, the present study conducted comparative analyses of mitogenome sequences from Korea, Vietnam, and Japan. The sequencing results indicated that *L. anatina* mitogenomes are extraordinarily longer than the typical animal mitogenome. Besides, the gene orders of *L. anatina* mitogenomes are variable among localities. The calculation based on protein-coding genes revealed relatively low substitution rates among mitogenome sequences from Buan (Korea), Doson (Vietnam) and Yanagawa (Japan). The phylogenetic analyses indicated the divergence of *L. anatina* in the western Pacific region. This investigation provides new information on the molecular systematics of *L. anatina* and could be helpful in exploring the diversity and evolution of mitogenomes in brachipod.

**Abstract:**

*Lingula anatina* is a brachiopod widely distributed in the western Pacific region. Even though *L. anatina* has been targeted for a number of biological studies, there is still limited information on intraspecific genetic variations of *L. anatina*. In this study, *L. anatina* specimens were collected from Korea and Vietnam, and complete mitochondrial genome (mitogenome) sequences were analyzed and compared with previous records. The total mitogenomes of *L. anatina* were 24,875 bp and 25,305 bp in size for Korean and Vietnamese specimens, respectively. Those mitogenomes are extraordinarily longer than the typical mitogenome size for an animal but shorter than the previous record from Yanagawa (Japan) for this species. The gene orders and the sizes of the protein-coding genes are also different from those for the Japanese specimen. Furthermore, the nonsynonymous (Ka) and synonymous (Ks) substitution rates in protein-coding genes (PCGs) were calculated to test the idea of evolutionary rate differences in mitochondrial genomes. The analyses showed relatively low Ka and Ks for the complete mitogenomes from Buan (Korea), Doson (Vietnam) and Yanagawa (Japan). The Ka/Ks ratio was less than 1 in comparisons of three localities, indicating the existence of purifying selection in this species. The phylogenetic analyses showed that *L. anatina* diverged among localities in the western Pacific region.

## 1. Introduction

Mitochondria are the energy production organelles of eukaryotic cells and have double-stranded circular genomes. Typically, mitochondrial genomes (mitogenome) are approximately 14 to 18 kb long in metazoans and contain 13 protein-coding genes, 22 transfer RNA (tRNA) genes, two ribosomal RNA (rRNA) genes and a control region [1]. Their categorization is based on several factors that have high variability, even at the family level, such as size, gene content, gene order, tRNA location and the replication of tRNAs [2,3]. These variable factors are phylogenetic markers [4,5,6,7]. Additionally, the stability of the mitogenome structure is variable. For instance, different from invertebrates, mitochondrial genomes in invertebrates such as Brachiopoda are more variable, even within species [8].

The phylum Brachiopoda consists of distinctive members of a marine invertebrate with approximately 404 extant species [9]. Having appeared in the early Cambrian period, this phylum has a long evolutionary history and a complete fossil record through extinction events on Earth [10]. Conservation characteristics and long geological records make brachiopods model animals for multiple studies, especially evolutionary systematics [11]. The studies on the evolutionary history of brachiopods are usually performed based on their fossil records, and a few studies are based on molecular approaches [12]. The application of molecular markers can provide new insights into the genetic diversity and historical processes of brachiopods [13]. *Lingula anatina* is widely found in the Indo-Western Pacific region and is one of the most studied organisms among the brachiopods [13,14]. There is a report that limited changes have occurred in *L. anatina* compared to its ancestor in the Cambrian period [15]. However, molecular markers, such as mitochondrial DNA and elongation factor 1α (*ef1α*) vary among sampling sites [13,14,16,17]. In this case, complete mitogenome analysis might provide more comprehensive information about genetic variation and the evolutionary history of *L. anatina*.

To date, a limited number of mitochondrial genomes of brachiopods have been sequenced and analyzed. For *L. anatina*, previous studies reported an unusual mitogenome of larger size, more extended repetitive sequences and more tRNA genes compared to other groups [18]. Additionally, comparisons of *L. anatina* mitogenome genes indicated high levels of variation among different localities [8,19]. This variation resulted in the speculation that there are cryptic species from various surveyed sites [8]. Therefore, further studies on the *Lingula* mitogenome will shed more light on mitogenome diversity, species identification and the evolution of this brachiopod. The present study aimed to investigate mitogenome variation and the evolutionary history of *L. anatina*. For this purpose, the complete mitogenomes of *L. anatina* from Korea and Vietnam were sequenced and compared to previous records from Korea and Japan. A phylogenetic tree was constructed based on mitogenome sequences to elucidate the relationships among *L. anatina* from different localities. Additionally, *cox1*-based phylogenetic analyses were performed to investigate the relationships of *L. anatina* from a broad geographical range in the western Pacific region. 

## 2. Results and Discussion

### 2.1. Mitogenome Structure

The mitogenomes lengths were 24,875 and 25,305 bp, and they were submitted to GenBank with accession numbers MW528457 and MH371361 for specimens from Buan, Korea, and Doson, Vietnam, respectively (Figure S1). The Buan mitogenome was almost identical to the previously recorded specimen from Incheon, Korea (KX774482). Only two complete mitogenomes belonging to *L. anatina* have been previously recorded from Incheon, Korea (KX774482) and Yanagawa, Japan (AB178773) that are 24,876 and 28,818 bp in length. One more *L. anatina* mitogenome was acquired from Amami Island, Japan (KP881498), but it was a partial genome (17,970 bp). All the mitogenome sequences examined to date have revealed that the mitogenome of *L. anatina* is extraordinary long for that of a metazoan. 

**Figure 1:**
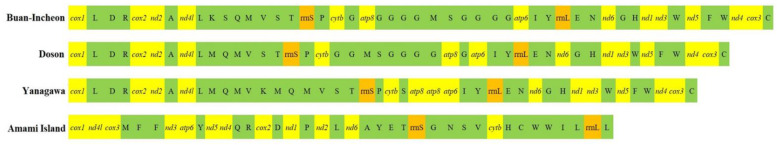
Gene organizations of *Lingula anatina* mitogenomes from Buan-Incheon (Korea), Doson (Vietnam), Yanagawa and Amami Island (Japan). Color indicators represent different groups: Yellow: Protein-coding genes, Green: tRNA genes, Orange: rRNA genes.

A typical metazoan mitogenome consists of 13 protein-coding genes (PCGs), 22 tRNA genes and two rRNA genes [1]. However, 13 PCGs, 34 tRNA genes and two rRNA genes were identified in the mitogenome of the Buan specimen (Table 1), and 13 PCGs, 31 tRNA genes and two rRNA genes were identified in the mitogenome of the Doson specimen (Table 2). The main difference between the two records was caused by the number of tRNA-Gly genes. While there were ten tRNA-Gly genes in the Buan specimen, there were eight genes in the Doson specimen. The nucleotide distribution of the *L. anatina* mitoge-nome was 26.3%, A, 21.3% G, 15.9 % C and 36.5% T for the Buan specimen. Meanwhile, the nucleotide distribution was 26.1% A, 21.5% G, 15.8% C and 36.6% T for the Doson specimen. These values are slightly different from those of the Yanagawa specimen, which contained 26.1% A, 21.9% G, 16.1% C and 35.9% T. The whole genes were encoded on the heavy strands of the two new mitogenomes, which is consistent with the previous records from Incheon, Korea and Yanagawa, Japan [18,19]. Although the number of coding genes was variable, relative orders of PCGs and two rRNA genes were similar in the mitogenomes of *L. anatina* from Buan, Incheon, Doson and Yanagawa, while the Amami Island mitogenome is different from these sequences. Overall, mitogenomes from Buan, Doson and Incheon had identical positions for the first nine genes, from *cox1* gene to tRNA-Leu (Figure 1). After tRNA-Leu, Doson and Yanagawa mitogenomes shared four more tRNA genes, including two tRNA-Met genes, tRNA-Val and tRNA-Gln. Similarly, if counted from the right direction (tRNA-Cys), gene positions were identical for these sequences from tRNA-Cys to *atp6*. The gene rearrangements occurred in the position between tRNA-Leu and *atp8* gene. A number of events such as duplication, translocation, insertion and deletion may result in this rearrangement (Figure 1). 

There were 45 intergenic sequences in the mitogenome of Buan specimen and 41 intergenic sequences in the mitogenome of the Doson specimen. The ranges of intergenic sequences varied from 1 to 1633 bp in the Buan specimen and from 1 to 1628 bp in the Doson specimen. On the other hand, there was no overlapping region identified within the two mitogenomes. The GC skewness of the mitogenomes was slightly positive for both new sequences of *L. anatina*, indicating a higher content of G than C; AT skewness had slightly negative values indicating a higher occurrence of T than A (Figure S2). This finding is consistent with the mitogenome sequence reported in the previous study [18,19]. 

### 2.2. Protein-Coding Genes

The PCGs of Buan and Doson *L. anatina* were 11,919 and 11,952 bp in length, constituting 47.9% and 47.2% of their total mitogenomes, respectively. By comparison, the relative rearrangements of PCG positions were similar in all the complete mitogenome records (MW528457, KX774482, MH371361 and AB178773,). However, although the Amami Island sequence KP881498 is incomplete, this record has 12 protein-coding genes (without *atp8*), and its gene order is dramatically different. The previous study already mentioned that this specimen could belong to a cryptic species [8]. The authors also suggested improving record numbers from different localities to resolve this unclear point. Our comparison between Buan, Doson and Yanagawa specimens revealed that there is no dramatic difference in PCG structures or orientations in *L. anatina* mitogenomes. However, the sizes of the PCGs were slightly variable among the *L. anatina* records (Table S1). 

**Figure 2:**
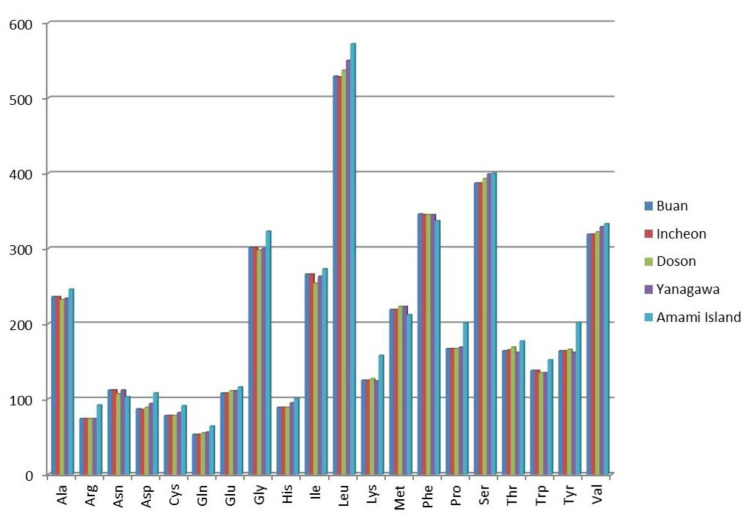
Amino acids corresponding to the codons of protein-coding genes of the *Lingula anatina* specimens. The specimens represented with localities: Buan, Korea (MW528457), Incheon, Korea (KX774482), Doson, Vietnam (MH371361), Yanagawa, Japan (AB178773) and Amami Island, Japan (KP881498).

Most of PCGs in the mitogenomes of the Buan and Doson specimens used AT- codons for initiation. Only *cox1* and *cox3* in the Buan specimen and *cox3* and *nd4l* in the Doson specimen used GTC as a start codon. TAA and TAG were only two common termination codons in the mitogenomes of *L. anatina*. In the Buan specimen, TAA was used by the genes *atp6, atp8, cox2*, *cox3*, *nd1* and *nd2,* while TAG was used by the genes *cox1, cytb*, *nd3*, *nd4*, *nd4l*, *nd5* and *nd6*. In the Doson specimen, TAA was used by the genes *atp6, atp8, cox1*, *cox3*, *nd1*, *nd2* and *nd4*, while TAG was used by the genes *cox2*, *cytb*, *nd4l*, *nd3*, *nd5* and *nd6*. The amino acids corresponding to the codons of PCGs were also analyzed (Figure 2). According to codon usage analysis, the most used amino acids were Leu and Ser, while the least-used amino acid was Gln.

Furthermore, the nonsynonymous (Ka) and synonymous (Ks) substitution rates in PCGs of the specimens were calculated to test the idea of evolutionary rate differences in mitochondrial genomes since the evolutionary rates of mitochondrial genomes have proven to be correlated with speciation rates [20,21,22]. Due to Yanagawa specimen having two *atp8* genes and Amami Island specimen having no *atp8* gene, this gene was excluded for the Ka-Ks substitution rate analysis. Additionally, two mitogenomes of Korean specimens were almost identical, the newly sequenced mitogenome from Buan was used for the calculation. The comparison among Buan, Doson and Yanagawa specimens showed that Ka values ranged from 0 to 0.087 and Ks values ranged from 0.032 to 1.098 (Table S2). In addition, Ka values between Doson and Yanagawa specimens were the smallest for all genes, except *nd4l* gene. The Ka values from these comparisons were smaller than the values obtained from the comparison between specimens from Yanagawa and Amami Island, Japan [8].

Comparing the complete mitogenome sequences from Buan and Doson to the mitogenome sequence (KP881498) from Amami Island, Japan showed high values for both Ka (0.059–0.531) and Ks (0.153–4.329) (Table S2). This is consistent with the analysis in a previous study that compared two mitogenomes of *L. anatina* collected from Yanagawa and Amami Island [8]. Luo et al. (2015) revealed that Ka values between these two specimens varied from 0.078 (*cox1*) to 0.357 (*nd5*). Additionally, compared to other invertebrate mitogenomes, these values were comparable to interspecific variation [8]. Due to the high Ka and Ks, the authors had doubts about the species identification and suggested that the specimen collected from Amami Island may belong to a different species. Our finding here supported the finding by Luo et al. (2015) and further study should be performed to confirm the existence of cryptic *L. anatina* from Amami Island. 

The calculation of Ka/Ks ratios among Buan, Doson and Yanagawa that all values were less than 1 for all three mitogenome comparisons (Table S2, Figure S3). The obtained results revealed purifying selection in *L. anatina* from these localities. Comparing Buan-Amami Island mitogenomes and Doson-Amami Island mitogenomes also showed a similar pattern, except for *cox2* and *nd4* genes with Ka > Ks (Table S2). 

### 2.3. Transfer RNAs and Ribosomal RNAs

There were 34 tRNA genes in *L. anatina* mitogenome from Buan that were 52–72 bp in length. For the *L. anatina* mitogenome from Doson, there were 31 tRNA genes with the absence of tRNA-Lys. tRNAs of the Doson mitogenome also ranged from 52 to 72 bp. The number of tRNAs was variable in the *L. anatina* records. The number of tRNA genes was 27 for the complete mitogenome from Yanagawa, Japan and 23 for the incomplete mitogenome from Amami Island, Japan. It is worth noting that the number of tRNA genes in the Yanagawa mitogenome is possibly higher than 27. In addition to 27 tRNA genes, previous study reported tRNA-like sequences in this mitogenome [18]. The search for tRNA genes of the Yanagawa mitogenome with the prediction program ARWEN suggested a number of tRNA-like sequences could be real tRNA genes [23].

Twenty-two tRNAs with repetition of tRNA-Leu and tRNA-Ser are seen in typical metazoan mitochondrial genomes [24]. The mitogenomes of *L. anatina* are atypical regarding tRNAs. All four records showed some variation in the repetition of tRNAs (Figure S1). In the Buan specimen, tRNA-Gly was repeated ten times, while in the Yanagawa specimen it was seen only one time. Besides, the number of tRNA-Trp and tRNA-Met in *L. anatina* mitogenome were also higher than those of typical animal mitogenome. In addition, tRNA structures also showed variety for the tRNA-Ser. Typically, the dihydrouridine arm of all the tRNAs is a large loop instead of a conserved stem structure in the metazoan mitochondrial genomes, excluding tRNA-Ser. In most tRNA-Ser for AGY/N codons lack the dihydrouridine arm in the metazoan [25,26,27,28]. However, the dihydrouridine arms were 3 to 4 bp long in the *L. anatina* mitogenomes. In addition, the amino acid arms and the TΨC arms varied in length from 4 to 5 bp in all of the analyzed *L. anatina* mitogenome tRNA genes.

There were two ribosomal RNA genes observed in the mitogenome of all the *L. anatina* specimens. They were all encoded on the major strand. The size of 16S rRNAs varied from 1299 to 1483 bp with 56.9% to 61.7% A–T content. The shortest 16S rRNA was observed in the incomplete mitogenome from Amami Island, Japan. Apart from this incomplete sequence, comparing only complete mitogenomes indicated that 16S rRNAs were almost identical, at 1462 to 1465 bp in length. In 12S rRNA, a similar pattern also occurs. The 12S rRNA of the complete mitogenomes of *L. anatina* were almost identical, at 1270 to 1271 bp in length. 

### 2.4. Non-Coding Region

Non-coding regions of the mitogenome were confirmed with primer sets listed in Table S3. Of 12 amplified sequences, nine sequences had similar sizes with NGS data, while three sequences had shorter sizes than NGS data (Table S3). The shorter sizes in obtained sequences were caused by incorrect repetitive elements in the assembly of NGS data. The final non-coding regions were assembled with sequences generated by PCR and Sanger sequencing. 

The comparison of the non-coding region among localities is presented in Table S4. The size, repeat and potential open reading frame in non-coding regions were variable for the mitogenomes from Korea, Vietnam and Japan. In total, there were 45 non-coding regions in the Buan mitogenome and 41 non-coding regions in the Doson mitogenome (Tables S5 and S6). The largest non-coding region of the Buan mitogenome was 1633 bp in length and located between tRNA-Met and tRNA-Val (Table S5). The largest non-coding region of the Doson mitogenome was 1628 bp in length and located between tRNA-Gln and tRNA-Met (Table S6). Meanwhile, the largest non-coding region of the Yanagawa mitogenome was up to 4017 bp and located between tRNA-Ser and *atp8* genes.

In total, six unassigned repeated sequences (URSs), were observed in the non-coding region of the Buan mitogenome with lengths from 31 to 223 bp (Table S7). Additionally, seven URSs were observed in the non-coding region of the Doson mitogenome, with lengths from 53 to 223 bp (Table S8). The numbers of URSs in the Buan and Doson mitogenomes are smaller than those in the Yanagawa mitogenome and the lengths of repeated sequences are also different (Table S4). The longest URS in the Yanagawa mitogenome was 1092 bp, whereas the longest in the Buan and Doson mitogenome was 223 bp. 

Similar to the Yanagawa mitogenome, the repeated sequences in Buan and Doson mitogenomes were mainly located in Repeat region I: *cox2*-tRNA-Val and Repeat region II: *cytb*-*atp6*. The details of comparison between the Buan and Doson mitogenomes are presented in Figure S4. In Repeat region I, even though each mitogenome contained two URSs, their locations are different. The pseudo-gene and tandem repeat observed in the Yanagawa mitogenome was not found in the Buan and Doson mitogenomes. In addition to URS, one and two unassigned unique sequences were found in Repeat region I of the Buan and Doson mitgenomes, respectively. Compared to Repeat region I, more URSs was observed in Repeat region II for both mitgenomes. There were four URSs in the Buan mitogenome and five URSs in the Doson mitogenomes observed in this. As shown in Figure S4, URSs in both mitogenomes were mostly located in the second half of Repeat region II. 

Additionally, a number of ORFs were found in the Buan and Doson mitogenomes (Tables S9 and S10). There were 15 ORFs in the Buan mitogenome that varied from 156 to 396 bp. The Doson mitogenome included 17 ORFs with the length of 153 to 492 bp. The translated amino acid sequences from ORFs are presented in Figure S5. ORFs in *L. anatina* may encode for amino acid sequences with unassigned function [18].

### 2.5. Phylogenetic Studies

**Figure 3:**
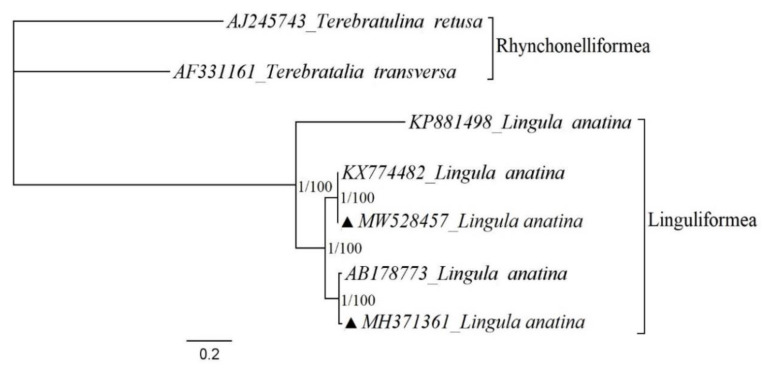
Phylogenetic relationships of *Lingula anatina* specimens from different localities based on mitochondrial protein-coding genes. The complete mitochondrial genomes for reconstruction of phylogenetic tree retrieved from GenBank and the subphylum Rhynchonelliformea records were chosen as representatives of the outgroup. The triangle shows the records sequenced for the present study. The numbers at the internodes are bootstrap values of Maximum likelihood (**right**) and posterior probability values of Bayesian inference (**left**).

Bayesian inference and maximum likelihood phylogenetic analyses were performed with five *L. anatina* and two Rhynchonelliformea as representatives of outgroup records (Figure 3). The results of two maximum likelihood and Bayesian inference analyses based on nucleotide datasets of 12 PCGs (*atp8* excluded) are similar (Figure 3). Additionally, analyses suggested that the Vietnamese specimen has a sister relationship with the Yanagawa specimen, and the closest relatives to this clade were two Korean specimens that are almost identical. The phylogenetic tree also indicated that the Korean specimens diverged early from the Doson and Yanagawa specimens. Additionally, phylogenetic analysis showed that although the Amami Island specimen has a relationship with other *L. anatina* specimens, the distance is far in comparison with the relationships of the other records. These findings may suggest that there is a cryptic species among complete mitogenomes of *L. anatina* [8].

In addition, 37 partial *cox1* sequences (MW519149-MW519185), including 25 specimens from Buan and 12 specimens from Incheon, were generated to investigate the relationships among *L. anatina* from different localities in the western Pacific region. The phylogenetic analyses suggested that the Korean specimens formed a cluster. These specimens were collected from two localities of the eastern side of the Yellow sea that were positioned close to a Chinese specimens. The phylogenetic tree also revealed that the Yanagawa specimen had a sister relationship with the Doson specimen (Figure S6). Meanwhile, Amami Island and Hong Kong specimens are distant from sequences of the remaining localities. The genetic separation of species is mainly explained by several historical and contemporary processes, such as genetic drift, natural selection, population isolation and range expansion [29,30]. In a previous study, two distinct lineages in *Scomber japonicus* populations representing Chinese and Japanese types were observed based on the *cytb* gene marker [30]. Future studies are necessary to clarify the mechanisms of genetic variations of *L. anatina* in the western Pacific region. 

The phylogenetic trees also revealed that specimens from different localities formed different clusters. This finding suggests distinct phylogeographic differentiation, and there are different groups of *L. anatina*. The genetic variations of *L. anatina* among localities were observed in previous studies based on several markers, such as *cox1*, *ef1α* and 18S rRNA [8,17,18]. Analyses of partial *cox1* and *ef1α* sequences demonstrated that genetic distances of *L. anatina* from different localities were relatively high and that there were close relationships between East and South China Sea specimens [18]. Our study supports the recent genetic studies that consider *L. anatina* to not be an unchanging species [13,18]. 

Both complete mitogenome and partial *cox1* gene-based analyses showed that the specimen from Amami Island is distant from the other *L. anatina* specimens. The pattern of phylogenetic trees is congruent with Ka and Ks analyses, which resulted in high Ka and Ks values when comparing the Buan, Doson and Yanagawa mitogenomes with the Amami Island mitogenome. Recent investigations showed that the breeding season of *L. anatina* at Amami Island is also different from those of other groups in Japan [31]. The evidence supported the suggestion that the Amami Island specimen is a cryptic species. Taxonomical study in the future study is required to clarify the species complex of *L. anatina*.

## 3. Material and Methods

### 3.1. Sampling and DNA Sequencing

**Figure 4:**
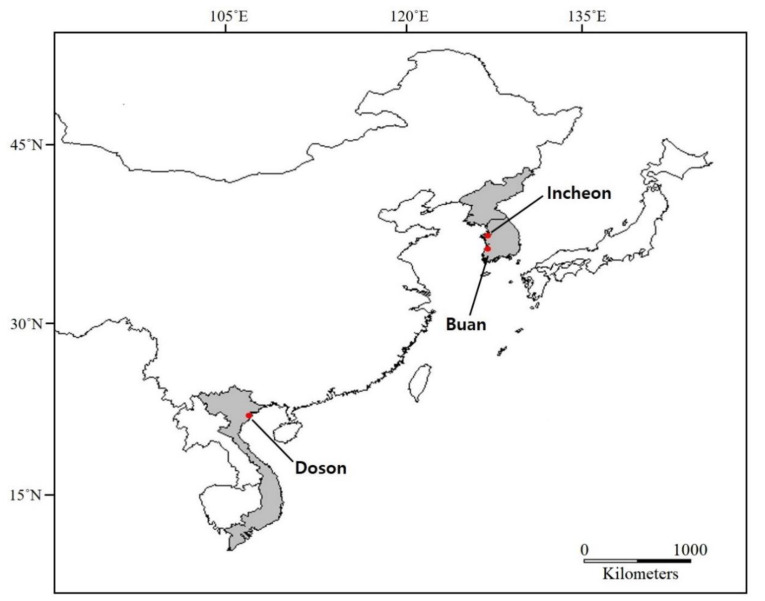
Map shows sampling localities in this study: Buan and Incheon (Korea) and Doson (Vietnam).

The *L. anatina* specimens were collected from Incheon, Korea (37°26′53″ N, 126°22′13″ E) in February 2016, Buan, Korea (35°34′55.87″ N, 126°30′52.56″ E) in July 2019) and Doson, Vietnam (20 46′16,22″ N, 106° 40′05,93″ E) in September 2016 (Figure 4). The collected specimens were deposited at the Department of Biotechnology, Sangmyung University, Korea University; 97% ethanol preservation. Procedures to obtain partial *cox1* sequences were performed following a previous study [16]. One individual from each Buan (Korea) and Doson (Vietnam) specimens was chosen for mitogenome sequencing. The Buan specimen was collected approximately 200 km away from the previously recorded Incheon specimen [19]. Briefly, the mitochondrial DNA was isolated using a mitochondrial DNA isolation kit (Biovision, Milpitas, CA, USA) according to manufacturer’s recommendations. Isolated DNA was measured for purity using the DropSense96 UV/VIS droplet reader (Trinean, Gentbrugge, Ghent, Belgium). The quantification of the DNA concentration was performed using Quanti-IT PicoGreen dsDNA kit (Invitrogen, Carlsbad, CA, USA), and the integrity of extracted DNA was verified using an agarose gel. A total of 200 ng of mitochondrial DNA was sheared using S220 Ultrasonicator (Covaris, Woburn, MA, USA). Library preparation was performed with an Illumina Truseq Nano DNA sample prep kit (Illumina, San Diego, CA, USA) following the manufacturer’s protocols. Finally, the quality and the band size of libraries were assessed using Agilent 2100 bioanalyzer (Agilent, Santa Clara, CA, USA). Libraries were quantified by qPCR using CFX96 Real-Time System (Bio-Rad, Hercules, CA, USA). After normalization, sequencing of the prepared library was conducted on Miseq system (Illumina, San Diego, CA, USA) with 300 bp paired-end reads. 

### 3.2. Analysis of Mitochondrial DNA

Mitochondrial genes were assembled and annotated by MITObim software and MITOS web server [32,33]. PCGs and rRNA genes were also confirmed by BLAST searches in GenBank by alignment with homologous genes from other *L. anatina* species. Transfer RNA genes were identified by comparing the results predicted by tRNAscan-SE Search Server v.1.21 and ARWEN based on cloverleaf secondary structure information [23,34]. The secondary structures of ribosomal RNAs were predicted by the LocARNA webserver [35]. The annotation of mitogenome sequences was manually refined using Geneious software version 9.1.2 [36]. 

Due to repeated sequences in non-coding regions of *L. anatina* mitogenome, 12 overlapping primer sets were designed to confirm these sequences (Table S3). The PCR mixture was prepared, containing 10 μL of 2× TOPsimple^TM^ DyeMIX-Tenuto (Enzynomics, Daejeon, Korea), 1 μL of each primer (10 pmoles/μL), 100 ng of DNA, and distilled water to make the mixture 20 μL. The amplification procedure was performed as: 95 °C for 5 min, followed by 35 cycles of denaturation at 95 °C for 1 min, annealing at 50 °C for 1 min, extension at 72 °C for 1 min, and final elongation at 72 °C for 5 min. The PCR products were run with electrophoresis in 1% agarose gels in 1× TAE buffer. Sequencing was conducted using an ABI 3730 DNA Analyzer (Applied Biosystems, Waltham, MA, USA). 

*L. anatina* mitogenome records from Korea and Japan were retrieved from GenBank for further analyses. Gene orders were reoriented as starting from *cox1* gene. Furthermore, non-synonymous (Ka) and synonymous (Ks) substitution rates were computed with the Ka/Ks calculator (v2.0) [37]. The repeated sequences and open reading frames were searched in Geneious software version 9.1.2 [36]. Unassigned unique sequence was searched for following previous study [18].

### 3.3. Phylogenetic Analyses

To better resolve the molecular phylogeny of *L. anatina*, the phylogenetic tree of *L. anatina* based on mitogenomes was reconstructed using previous records and two new sequences, including MW528457 (Buan, Korea), KX774482 (Incheon, Korea), MH371361 (Doson, Vietnam), AB178773 (Yanagawa, Japan), KP881498 (Amami Island, Japan). Among them, only KP881498 is not a complete mitochondrial genome, and these are the entire mitogenome records belonging to the subphylum Linguliformea. Additionally, two mitogenomes from rhynchonelliformean species, *Terebratalia transversa* (AF331161) and *Terebratulina retusa* (AJ245743) were selected as an outgroup. Since the *atp8* gene is not stable in the records, it has been excluded, and only sequences of 12 protein-coding genes were used in phylogenetic analysis. For further investigation, partial *cox1* sequences from *L. anatina* specimens were generated to investigate the *cox1*-based phylogenetic relationships. Furthermore, *cox1* sequences of *Lingula* species were retrieved from GenBank for analysis. The best fit model for the *cox1* dataset was searched with jModeltest [38]. Meanwhile, the model and partitioning of the complete mitogenome dataset were determined with Partition Finder 2 [39]. The phylogenies were reconstructed with the Bayesian inference method using Mrbayes version 3.2.7a [40] and the maximum likelihood method using RAxML version 8.0.0 [41]. For maximum likelihood method, the GTR + G model was used for each analysis and analyzed with 1000 bootstrap replicates. For the Bayesian inference method, four simultaneous Markov chains were run for ten million generations, with tree sampling occurring every 100 generations and a burn-in of 25% of the trees. The convergence of chain was implied by ESS > 200 in Tracer version 1.7 [42]. The obtained tree was visualized in FigTree v.1.4.3 [43].

## 4. Conclusions

In this study, the complete mitogenomes of *L*. *anatina* collected from Korea and Vietnam were sequenced to investigate mitogenome structures and genetic variations of the species in the western Pacific region. The mitogenomes of *L*. *anatina* are variable in gene order, sizes of PCGs, and the number of tRNAs. Phylogenetic trees based on PCGs of mitogenomes and partial *cox1* sequences showed that *L. anatina* was divided into distinct clades, and the most distant clade was the incomplete mitogenome from Amami Island, Japan. This result is consistent with a previous investigation of *L. anatina* among various geographical localities. In future studies, broader geographical collection and additional genetic makers are recommended to provide comprehensive genetic variations of *L. anatina*. 

## Figures and Tables

**Table 1:** Structure and orientations of *Lingula anatina* complete mitochondrial genome genes collected from Buan, Korea (MW528457).

Gene	Position		Size (bp)	Initiation Codon	Termination Codon	Intergenic Sequence	Strand
*cox1*	1	1722	1722	GTG	TAG	5	+
tRNA-Leu	1731	1798	68			8	+
tRNA-Asp	1802	1869	68			3	+
tRNA-Arg	1875	1942	68			5	+
*cox2*	1944	2693	750	ATG	TAA	1	+
*nd2*	2999	4036	1038	ATG	TAA	305	+
tRNA-Ala	4039	4104	66			2	+
*nd4l*	4109	4444	336	ATG	TAG	4	+
tRNA-Leu	4457	4523	67			12	+
tRNA-Lys	4808	4877	70			284	+
tRNA-Ser	4891	4960	70			13	+
tRNA-Gln	5036	5102	67			75	+
tRNA-Met	6736	6807	72			1633	+
tRNA-Val	6838	6904	67			30	+
tRNA-Ser	7425	7476	52			520	
tRNA-Thr	7625	7693	69			148	+
12S rRNA	7694	8964	1271			0	+
tRNA-Pro	8965	9033	69			0	+
*cytb*	9107	10,363	1257	ATG	TAG	73	+
tRNA-Gly	10,609	10,672	64			245	+
*atp8*	10,675	10,851	177	ATG	TAA	2	+
tRNA-Gly	11,069	11,135	67			217	+
tRNA-Gly	11,627	11,693	67			491	+
tRNA-Gly	12,189	12,255	67			495	+
tRNA-Gly	12,733	12,799	67			477	+
tRNA-Met	13,171	13,236	66			371	+
tRNA-Ser	13,241	13,308	68			4	+
tRNA-Gly	13,328	13,395	68			19	+
tRNA-Gly	14,265	14,332	68			869	+
tRNA-Gly	14,880	14,947	68			547	+
tRNA-Gly	15,475	15,541	67			527	+
*atp6*	15,547	16,275	729	ATG	TAA	5	+
tRNA-Ile	16,362	16,432	71			86	+
tRNA-Tyr	16,437	16,503	67			4	+
16S rRNA	16,504	17,966	1463			0	+
tRNA-Glu	17,968	18,035	68			1	+
tRNA-Asn	18,050	18,119	70			14	+
*nd6*	18,123	18,728	606	ATG	TAG	3	+
tRNA-Gly	18,734	18,800	67			5	+
tRNA-His	18,816	18,880	65			15	+
*nd1*	18,931	19,839	909	ATA	TAA	50	+
*nd3*	19,931	20,428	498	ATG	TAG	91	+
tRNA-Trp	20,503	20,571	69			74	+
*nd5*	20,582	22,330	1749	ATT	TAG	10	+
tRNA-Phe	22,331	22,395	65			0	+
tRNA-Trp	22,412	22,480	69			16	+
*nd4*	22,495	23,766	1272	ATA	TAG	14	+
*cox3*	23,915	24,790	876	GTG	TAA	148	+
tRNA-Cys	24,803	24,870	68			12	+

**Table 2:** Structure and orientations of *Lingula anatina* complete mitochondrial genome genes collected from Doson, Vietnam (MH371361).

Gene	Position		Size (bp)	Initiation Codon	Termination Codon	Intergenic Sequence	Strand
*cox1*	1	1725	1725	ATA	TAA	5	+
tRNA-Leu	1731	1798	68			5	+
tRNA-Asp	1802	1868	67			3	+
tRNA-Arg	1872	1939	68			3	+
*cox2*	1941	2690	750	ATG	TAG	1	+
*nd2*	2996	4033	1038	ATG	TAA	305	+
tRNA-Ala	4944	5009	66			910	+
*nd4l*	5013	5348	336	GTG	TAG	3	+
tRNA-Leu	5361	5427	67			12	+
tRNA-Met	5463	5534	72			35	+
tRNA-Gln	5542	5609	68			7	+
tRNA-Met	7238	7309	72			1628	+
tRNA-Val	7338	7404	67			28	+
tRNA-Ser	7925	7976	52			520	+
tRNA-Thr	8125	8193	69			148	+
12S rRNA	8194	9463	1270			0	+
tRNA-Pro	9464	9532	69			0	+
*cytb*	9608	10,864	1257	ATG	TAG	75	+
tRNA-Gly	10,878	10,944	67			13	+
tRNA-Gly	11,455	11,521	67			510	+
tRNA-Met	11,900	11,965	66			378	+
tRNA-Ser	11,970	12,037	68			4	+
tRNA-Gly	12,057	12,123	67			19	+
tRNA-Gly	12,615	12,682	68			491	+
tRNA-Gly	14,048	14,115	68			1365	+
tRNA-Gly	15,471	15,538	68			1355	+
*atp8*	15,543	15,719	177	ATG	TAA	4	+
tRNA-Gly	15,965	16,032	68			245	+
*atp6*	16,038	16,766	729	ATG	TAA	5	+
tRNA-Ile	16,793	16,863	71			26	+
tRNA-Tyr	16,868	16,934	67			4	+
16S rRNA	16,935	18,397	1463			0	+
tRNA-Glu	18,398	18,465	68			0	+
tRNA-Asn	18,480	18,549	70			14	+
*nd6*	18,555	19,160	606	ATG	TAG	5	+
tRNA-Gly	19,166	19,232	67			5	+
tRNA-His	19,250	19,314	65			17	+
*nd1*	19,335	20,273	939	ATA	TAA	20	+
*nd3*	20,365	20,862	498	ATG	TAG	91	+
tRNA-Trp	20,937	21,005	69			74	+
*nd5*	21,017	22,765	1749	ATT	TAG	11	+
tRNA-Phe	22,766	22,830	65			0	+
tRNA-Trp	22,845	22,913	69			14	+
*nd4*	22,928	24,199	1272	ATG	TAA	14	+
*cox3*	24,348	25,223	876	GTG	TAA	148	+
tRNA-Cys	25,232	25,300	69			8	+

## Data Availability

The data presented in this study are available in NCBI GenBank (Accession number: MW528457 and MH371361).

## Supplementary Materials

The following are available online at https://www.mdpi.com/article/10.3390/biology10050367/s1, Figure S1: Mitochondrial genomes of *Lingula anatina* collected from Buan, Korea (A) and Doson, Vietnam (B), Figure S2: AT and GC skew values in the mitochondrial genome records of *Lingula anatina* specimens, Figure S3: Calculations of nonsynonymous and synonymous Ka/Ks ratio of protein-coding genes of *Lingula anatina* specimens from Buan, Doson and Yanagawa, Figure S4: Structures of Repeat regions I and II of *Lingula anatiana* mitogenomes from Buan and Doson, Figure S5. Amino acid sequences encoded by unassigned open reading frames in non-coding regions of the Buan mitogenome (BO1-BO15) and Doson mitogenome (DO1-DO17), Figure S6: Phylogenetic relationships of *Lingula* species based on partial sequences of the mitochondrial *cox1* genes, Table S1: Protein-coding gene sizes in the *Lingula anatina* specimens, Table S2. The nonsynonymous and synonymous substitutions (Ka and Ks) estimated based on 12 protein-coding genes of *Lingula anatina* from Korea, Vietnam and Japan, Table S3: Primers and PCR products used for confirmation of non-coding regions of *Lingula anatina* from Buan, Korea, Table S4: Comparison of non-coding regions of *Lingula anatina* mitogenomes from different localities, Table S5: The non-coding regions of *Lingula anatina* from Buan, Table S6: The non-coding regions of *Lingula anatina* from Doson, Table S7: Unassigned repeated sequences found in non-coding regions of *Lingula anatina* from Buan, Korea, Table S8: Unassigned repeated sequences found in non-coding regions of *Lingula anatina* from Doson, Vietnam, Table S9: Unassigned open reading frames (≥50 aa) found in non-coding regions of *Lingula anatina* from Buan, Korea, Table S10: Unassigned open reading frames (≥50 aa) found in non-coding regions of *Lingula anatina* from Doson, Vietnam.
