# Supplementary material for: An Investigation of the Variations in Complete Mitochondrial Genomes of Lingula anatina in the Western Pacific Region
Source: Biology (Basel). 2021 Apr 25;10(5):367. doi: 10.3390/biology10050367 (PMC8146825; doi:10.3390/biology10050367)
Supplement: Supplementary file 1 [file biology-10-00367-s001.zip › Suplementary data_Figure.pdf]

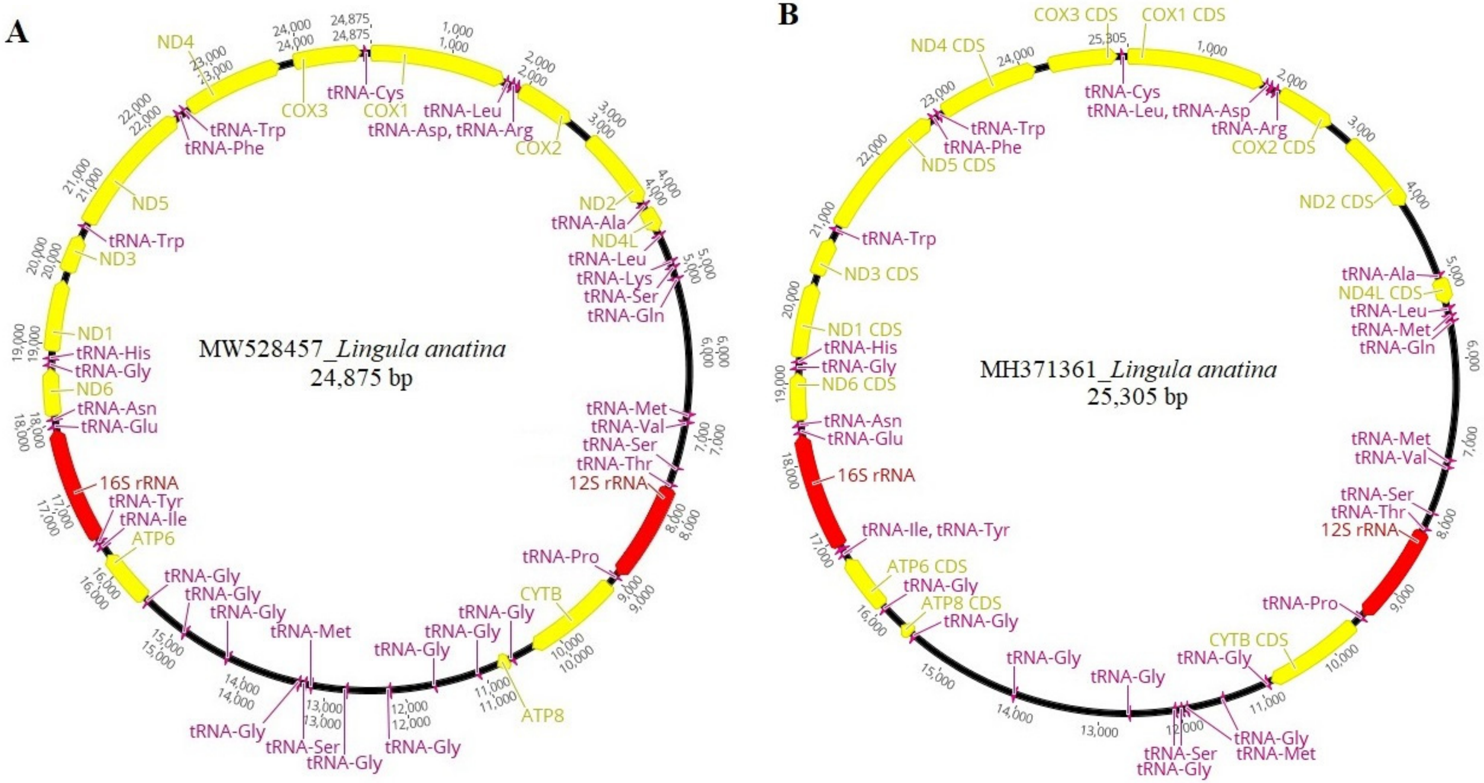

Figure S1. Mitochondrial genomes of the *Lingula anatina* collected from Buan, Korea (A) and Doson, Vietnam (B). Circular maps were drawn with the software Geneious v. 9.1.2. The arrows indicate the orientation of gene transcription. Abbreviations of gene names are: ATP6 and ATP8 for ATP synthase subunits 6 and 8; COX1–3 for Cytochrome oxidase subunits 1–3; CYTB for Cytochrome b, ND1–6 and ND4L for NADH dehydrogenase subunits 1–6 and 4l; and tRNA genes are indicated with their three-letter corresponding amino acids.

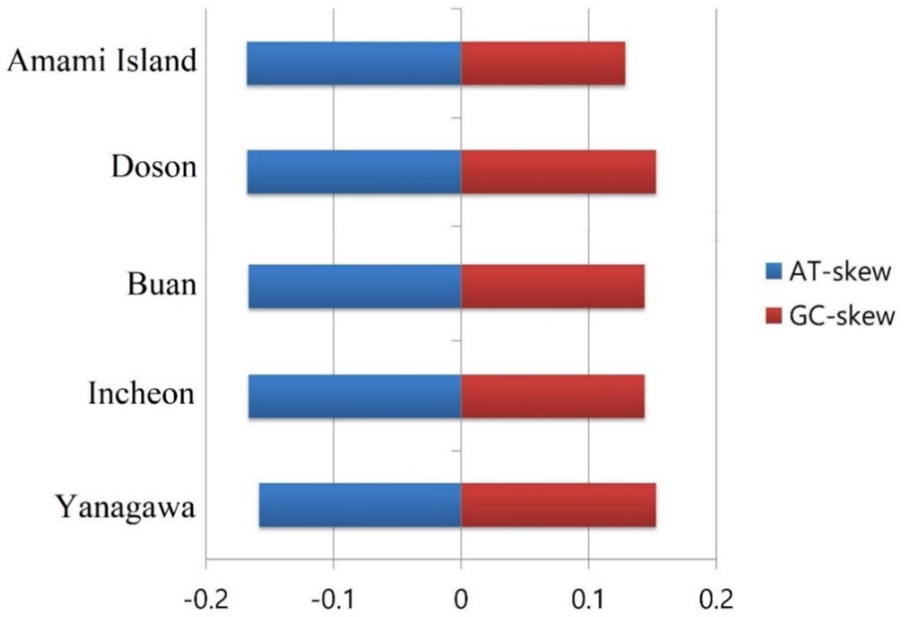

Figure S2. AT and GC skew values in the mitochondrial genome records of *Lingula anatina* specimens. The skewness was determined with a base composition of nucleotide sequences by using the formulae: AT skew = [A – T]/[A + T]; GC skew = [G – C]/[G + C]. The specimens are represented with localities: Buan, Korea (MW528457), Incheon, Korea (KX774482), Doson, Vietnam (MH371361), Yanagawa, Japan (AB178773) and Amami Island, Japan (KP881498).

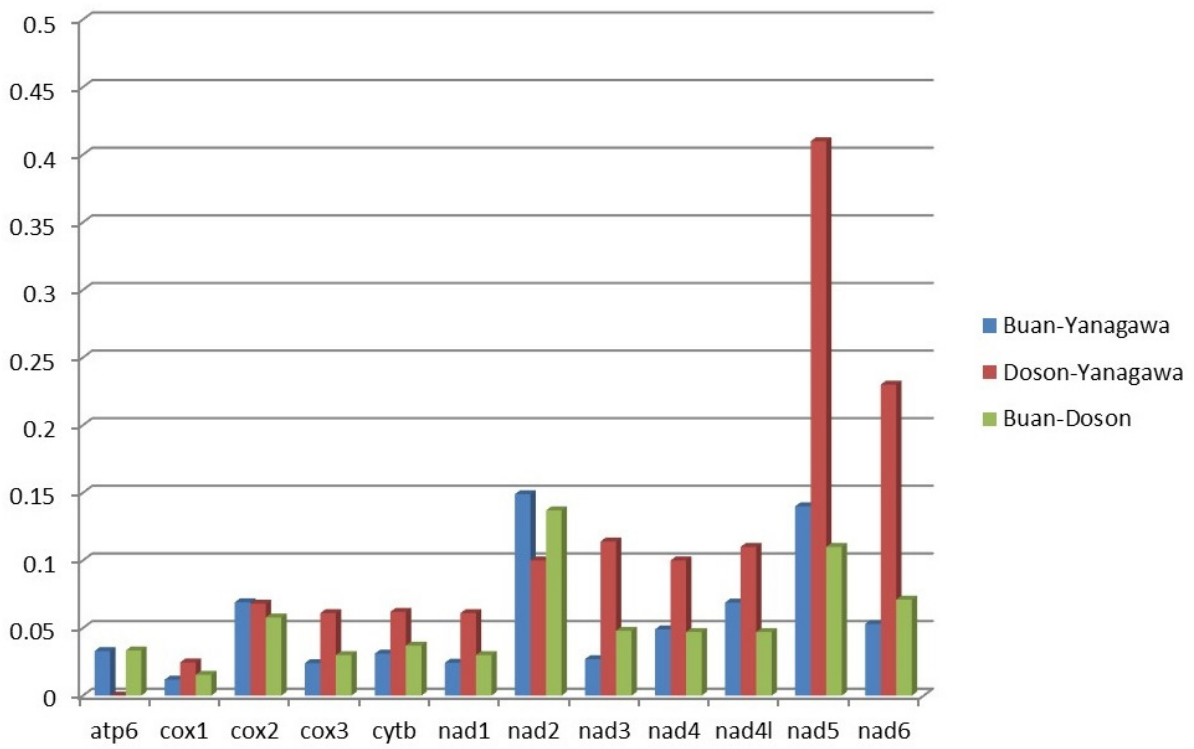

Figure S3. Calculations of nonsynonymous and synonymous Ka/Ks ratio of protein-coding genes of *Lingula anatina* specimens. For Ka/Ks calculation, protein-coding genes were compared between Buan, Korea (MW528457)-Yanagawa, Japan (AB178773), Doson, Vietnam (MH371361)-Yanagawa, Japan (AB178773), Buan, Korea (MW528457)-Doson, Vietnam (MH371361).

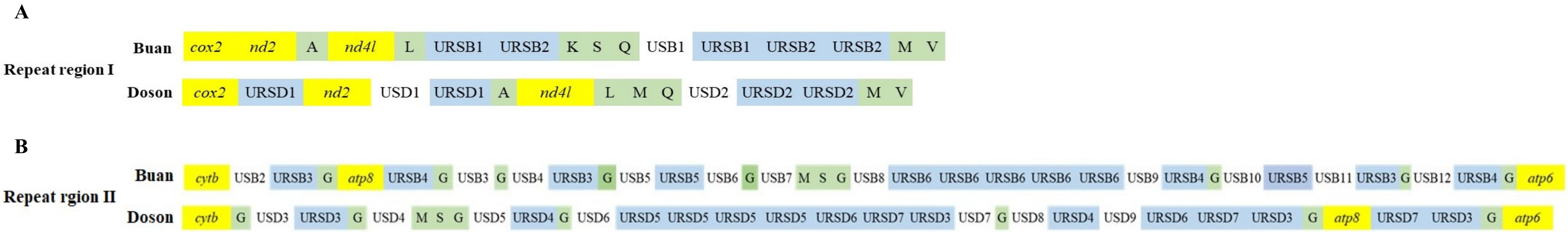

Figure S4. Structures of Repeat regions I and II of *Lingula anatiana* mitogenomes from Buan and Doson. A: Repeat region I and B: Repeat Region II. Abbreviations are as follows: URSB1–6: unassigned repeated sequence 1–6 of the Buan mitogenome; URSD1–7: unassigned repeated sequence 1–7 of the Doson mitogenome; USB1–12, unassigned unique sequence 1–12 of the Buan mitogenome: USD1–9: unassigned unique sequence 1–9 of the Doson mitogenome.

URFB1
MSCESLTSHLSYSSPSFLISGSDGAVDTALVVGPLSLGPLAGIQWSSSPLLDPPRPPPPFMGVGGGGPPLFGWKSPTLDTCFECLNSLPPRVGVKHSKQSIPMY
IWSSQIQDPDLGLWQASYTSHL
URFB2
MVIWLNKFKNKDLKPPYPQKFHFSLYSGKHPASILRGAPLDKSYIFHPGPP
URFB3
MKFLGVWGLKVLIFKFIKSNHHLSMFNLTKFKLNYMYLTGVPPLFTGGRGGKYTSYPEGPPVSWTPDVYLYMEKNEIFGGMGA
URFB4
MVIWLNKFKNKDLKPPYPQKFHFSLYSGKHPASILRGAPLDKSYIFHPGPP
URFB5
MPTIGPCLFWFFSSFIVFGLVYLFFMSFIQGQSCKKNWPVKLNFSRMGTNFRKSVSK
URFB6
MKNFNRAVVMKMMIGSFFFFFFSLMTKGYFMPYMMSWFILWFIKPMVDPVVYNKLRIFISGLCLVTLVLSWFFIPIDYIFDDNLKRFMKSLMGWMKLSSA
SELSNLGEHCLGELDASKFKK
URFB7
MEDFGWNFMRKVLIHSFFLFIFGWMTGGYLLHFMLGGLTLVFVKPLVDQEVYEKLRIFIYGMCVVISFVYWFFIPIDFIFDEYMKCFLDSLMDEIKLINSEMP
NFDEPCSCSPCFSEAKK
URFB8
MKKIDFNFIVKVVLNLLVFGLMINWTNGLFLPMYMGGFILYFVKPMVDLETYYKIMIFIMCMCFAVTMIYWLFVPVEFVFFPEIKCSRPEPSYSLNSVYHAI
KSIIYIK
URFB9
MWRSTHEFLKKVVSYCTVFSVFTLMLPPSMPIVFSVVILKFIQPLIDDPKTFNKIQIFVVCMCIVTYILFIFFIPLEFPVE
URFB10
MPEAGAVMPAAELLQPEPIVVEGGPLPEAGAVMPAAELLQPEPIVVEGGPLPEAGAVMPAAELLQPEPIVVEGGPLPEAGAVMPAAELLQPEPIVVEGGPLPE
AGAVMPAAELLQPEPIVVEGGPLPEAGA
URFB11
MPEFDFKFIVSVLSNLIVFGLLVHMSNGLFLPVFLGGFILYFVKPLVSLEVYYSLMIFIMIMGFMVFILNWLFFPSNFDVYVKFPVCYQERKYYLYSIYKEIES
FIYS
URFB12
MFMLSFQCVIKNENTIYTQFMKKLSLSFILNKSYKTLNPVKIEDILAMIGPCLFWFFSSFIVFGLVYLFFMSFIQGQSCKKNWPVKLDFSRMGTNFRKSVSK
URFB13
MSNRQQDYIFALCYFIFSLFYMWFIIEFTYGIMALMEWFSTADSSLVQYGCLQGDKIIPTVVCEGFPPLAKEDLPMSGYEQFLPLIEPECLKIKKPLPEIPVIVA

AFAK
URFB14
MSMDYFAKAATMTGISGSGFLILSHSGSIKGKNCSYPLMGSSSLAKGGNPSHTTVGMILSPWSHPYWTKLLSAVLNHSINAMIPYVNSMMNHM
URFB15
MGVFSVMKLSLQLCVKDFPLWLKKTFLWVGMSNFCLWLSLNVLSLSSPFLKFLLLLRLLQNNLWT

URFD1
MKTLDNYKHKSNNLPLAFDYYWDKQVKFAGKSVTLRVWKARTDSSEQESSTLKASLWVIVLMVGKFMLKVGVFLLLIWFGFFLGKNEKAYKSFSLISKK
YEYCFSIQKYADKVPKWLFFTAFYSVSVIAITCHVVIEYNFKTGTLRIESEGFMHRFKKNE
URFD2
MASNSNHRNPMECCKEKPLSHLISMLLNTKTMLMFLSNKSKSFVCLLIFTKKKTKSN
URFD3
MSSHTKDFLLFLSNMSIVLVFKSMLMSCLSGFSLQHSMGFRWLLLLAMSWLNMILKLELFVSSLKDLCTVLSSMSSL
URFD4
MLCFKCFTPTRGGSELSHLKQVSNVGDFHPKSGGPPAPYPHKGRGGAGGV
URFD5
MSCEALTSHLSYSSPSSLISGSDGAVDTALVVGPLSLGPLAGIQWSSSPLLDPPRPPPPFMGVGGGGPPAFWVEISYIGYLL
URFD6
MMIWLKSFKNKDHKPPYPQKFHFSLYSGKHPASILRGAPLDKSYIFHPGPP
URFD7
MEDFGWNFMRKVIIHGLFLFLFGWMSGGFFLHFMLGGLTLVFVKPLVDEDTYNKLRILIYGMCMVIGFMYWFFIPIDFIFDEYMKCFIDSLMDGIKLINSEV
PSLDEPCSCGPCFPEPKK
URFD8
MPKLGFEFYLKVVWYCFMLSGATYPLPVLFPVVFVVLSLNFIQPLVSDEVFGKMQVFSMAMCFTTIFIFGFLIPIEFIDD
URFD9
MKSLTRVVVMKMVVGSFFFFFFSFMTKGYFMPYMMSWFILWFIKPLVDPVVYSKLRIFISGLCLVTLVLYWFFIPIDYIFDDDLKRFIKSLMKWIKSSSGSEL
SNLGEHCLGELDDSKINK
URFD10
MSNWRFSMLMVLISFTFLSFIIWEFVDVTETLNLFVELVKEYTTNHHRTSVILPEVEFGATAGELTSPKFLQPEAIVVEGGPLSEVEAVLPEAEFLQPEAIVVE
GGPLSEVEAVLPEAEFPQPEAIVVEGGPLSEVEAVLPEAEFPQPEAIVVEGGPLSEVEA
URFD11
MEFWCRLGFSSISYLSSLKSTHAKAANLMNYSHRHSEKWKKSLPLMNNFQMPLFFHPLLLVFTMKVRKSAPTREKFNFTGQFFLQDWPWMKHKKKMNT
LSQKQWMS

URFD12
MSKFDLNFIIKVVLNLLVFGLIIDYTNGLFLPMYMGGLILYFVKPMVDLETYYKIMIFIMCMCFVVTMIYWLFVPAEFVFFPEIKCSRPEPSYTLNSVFHAIKS
IIYIK
URFD13
MSPKWQELIFAVSYLIFSLFYTWLVMECTCGIMMLFEWFNSADTSCLAQCGALQGDEVVPSAVCEGSPSLIKENTPMPENGEFLPCPEPECFKVKERIPEIPV
LAAAFAQ
URFD14
MLSMQIYCAKAAASTGISGMRSLTLKHSGSGQGKNSPFSGMGVFSLIKEGDPSHTAEGTTSSPWSAPHWAKQLVSALLNHSNNIMIPQVHSMTSHV
URFD15
MKLFLQLCVKDLLLWLKKMLLYLSMESSCLVLSLSVSSLSSAFLKFLFLLRLLHSKSVYSTFLVKFFSCWNCLPTPLWQN
URFD16
MVEFDLKFIVSVLSNLIVFGLLVDMSNGLFLPVFLGGFVLCFIKPLVSLEVYYSLLIFIMIMSFMVFILNWLFFPSNFDVYVSFPVCYQEPKYYLYSICKEIKSFI
YA
URFD17
MFMLGSQCVIKNQNTIYTQFAKKLSLSFMLNKFYKTLSLVKIEDIWPTIGSYKWKKNWKVCCLSAELVIFYFFSLMPTIGPCLFWFFSSFIVFGLVYLFFLCFI
QGQSCKKNWPVKLNFSRVGTDFRTFMVKTNNKGWKNNGI

Figure S5. Amino acid sequences encoded by unassigned open reading frames in non-coding regions of the Buan mitogenome (URFB1-URFB15) and Doson mitogenome (URFD1-URFD17).

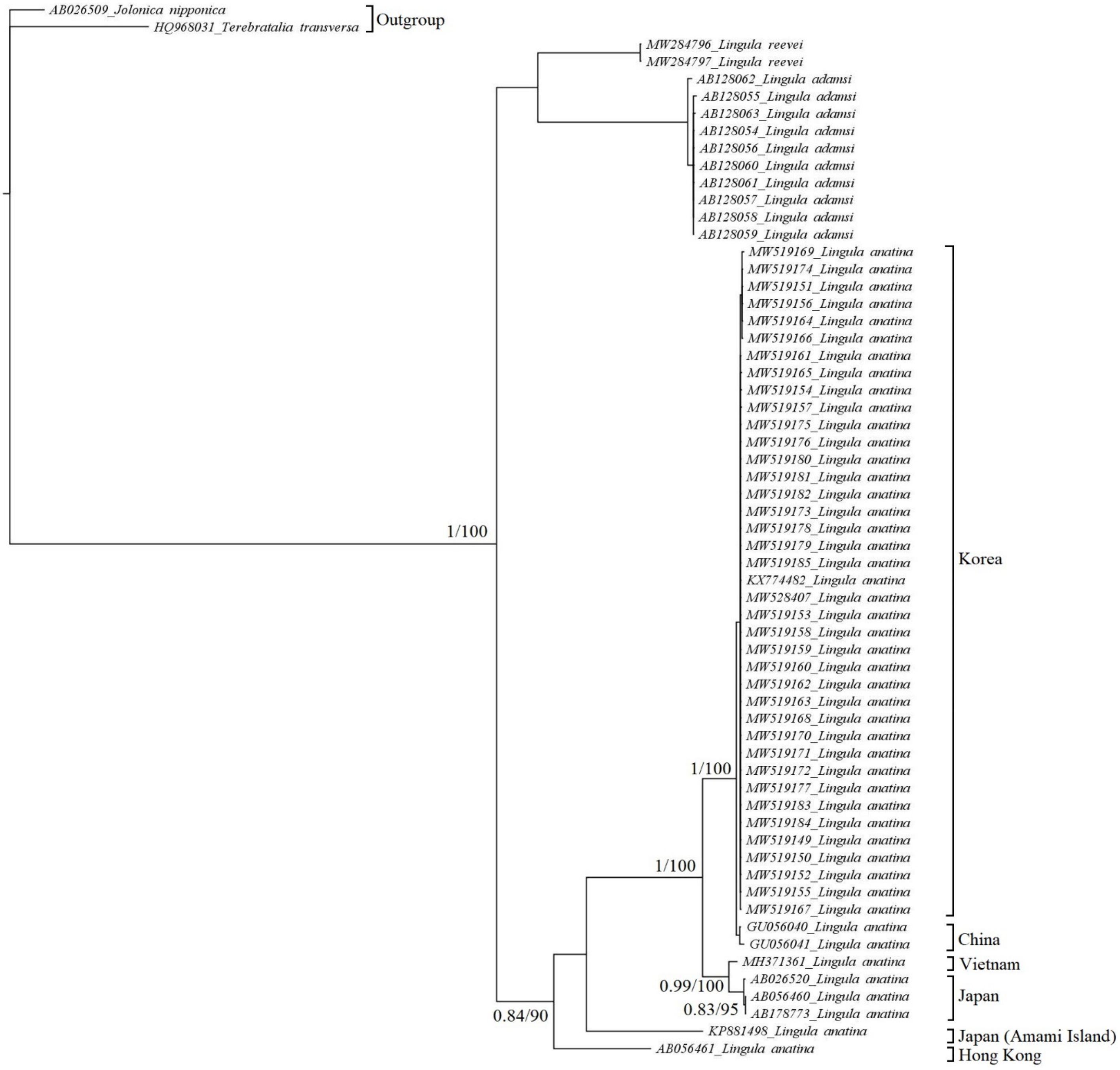

Figure S6. Phylogenetic relationships of *Lingula* species based on partial sequences of the mitochondrial *cox1* gene. Bootstrap support value (left) > 70 and posterior probability value (right) > 0.7 (left) are shown at nodes.
