# Supplementary material for: An Investigation of the Variations in Complete Mitochondrial Genomes of Lingula anatina in the Western Pacific Region
Source: Biology (Basel). 2021 Apr 25;10(5):367. doi: 10.3390/biology10050367 (PMC8146825; doi:10.3390/biology10050367)
Supplement: Supplementary file 1 [file biology-10-00367-s001.zip › Suplementary data_Table.pdf]

**Table S1.** Protein-coding gene sizes in *Lingula anatina* specimens. The *atp8* gene is duplicated in the Yanagawa mitogenome, while the same gene was not identified in the Amami Island mitogenome.

| Gene | Buan, Korea (MW528457) (bp) | Incheon, Korea (KX774482) (bp) | Doson, Vietnam (MH371361) (bp) | Yanagawa, Japan (AB178773) (bp) | Amami Island, Japan, (KP881498, Incomplete) (bp) |
|---|---|---|---|---|---|
| ***atp6*** | 729 | 729 | 729 | 729 | 792 |
| ***atp8*** | 177 | 177 | 177 | 177*2 | - |
| ***cox1*** | 1,722 | 1,722 | 1,725 | 1,722 | 1,599 |
| ***cox2*** | 750 | 750 | 750 | 750 | 741 |
| ***cox3*** | 876 | 876 | 876 | 828 | 1,008 |
| ***cytb*** | 1,257 | 1,257 | 1,257 | 1,257 | 1,302 |
| ***nd1*** | 909 | 909 | 939 | 957 | 1,014 |
| ***nd2*** | 1,038 | 1,038 | 1,038 | 1,041 | 1,005 |
| ***nd3*** | 498 | 498 | 498 | 498 | 549 |
| ***nd4*** | 1,272 | 1272 | 1,272 | 1,272 | 1,344 |
| ***nd4l*** | 336 | 336 | 336 | 336 | 291 |
| ***nd5*** | 1,749 | 1,749 | 1,749 | 1,749 | 1,788 |
| ***nd6*** | 606 | 606 | 606 | 606 | 597 |

**Table S2.** The nonsynonymous and synonymous substitutions (Ka and Ks) estimated based on 12 protein-coding genes of *Lingula anatina* from Korea, Vietnam and Japan.

| Gene | Buan-Yangawa (MW528457-AB178773) | | Doson-Yanagawa (MH371361- AB178773) | | Buan-Doson (MW528457- MH371361) | | Buan-Amami Island (MW528457-KP881498) | | Doson-Amami Island (MH371361-KP881498) | |
|---|---|---|---|---|---|---|---|---|---|---|
| | **Ka** | **Ks** | **Ka** | **Ks** | **Ka** | **Ks** | **Ka** | **Ks** | **Ka** | **Ks** |
| ***atp6*** | 0.037 | 1.098 | 0.000 | 0.038 | 0.037 | 1.077 | 0.147 | 3.638 | 0.135 | 3.607 |
| ***cox1*** | 0.010 | 0.813 | 0.002 | 0.093 | 0.011 | 0.687 | 0.075 | 4.324 | 0.059 | 4.329 |
| ***cox2*** | 0.017 | 0.249 | 0.005 | 0.072 | 0.015 | 0.264 | 0.407 | 0.208 | 0.384 | 0.248 |
| ***cox3*** | 0.012 | 0.463 | 0.011 | 0.108 | 0.017 | 0.548 | 0.1850 | 3.835 | 0.188 | 3.867 |
| ***cytb*** | 0.016 | 0.501 | 0.002 | 0.032 | 0.018 | 0.480 | 0.133 | 3.749 | 0.118 | 4.071 |
| ***nd1*** | 0.012 | 0.507 | 0.002 | 0.032 | 0.015 | 0.481 | 0.113 | 3.919 | 0.105 | 3.878 |
| ***nd2*** | 0.045 | 0.303 | 0.009 | 0.086 | 0.045 | 0.330 | 0.216 | 2.063 | 0.223 | 4.083 |
| ***nd3*** | 0.021 | 0.763 | 0.010 | 0.091 | 0.032 | 0.661 | 0.203 | 3.453 | 0.229 | 3.495 |
| ***nd4*** | 0.038 | 0.774 | 0.011 | 0.098 | 0.037 | 0.773 | 0.531 | 0.153 | 0.484 | 0.305 |
| ***nd4l*** | 0.029 | 0.545 | 0.034 | 0.106 | 0.041 | 0.506 | 0.186 | 3.106 | 0.211 | 1.929 |
| ***nd5*** | 0.087 | 0.764 | 0.056 | 0.136 | 0.080 | 0.728 | 0.462 | 0.725 | 0.376 | 2.114 |
| ***nd6*** | 0.034 | 0.635 | 0.010 | 0.044 | 0.041 | 0.575 | 0.121 | 2.410 | 0.129 | 2.217 |

**Table S3.** Primers and PCR products used for confirmation of non-coding regions of *Lingula anatina* from Buan, Korea.

| No. | Primer name | Sequence (5'-3) | Binding site* | Primer size (bp) | Expected size (bp) | PCR amplification size (bp) |
|---|---|---|---|---|---|---|
| 1 | La1-F | TCGTTGGTCAGGGTCTGATAAC | 4,393-4,114 | 22 | 438 | 438 |
| | La1-R | AGGAGGTGGTTTATTAGCCACT | 4,853-4,874 | 22 | | |
| 2 | La2-F | AGAATATAAAGTCCCATCTTTTG | 4,553-4,575 | 23 | 838 | 838 |
| | La2-R | GGATCCAATGATCTAGGAGGCC | 5,412-5,433 | 22 | | |
| 3 | La3-F | GAGATTTCCACCCAAAAAGCGG | 5,330-5,351 | 22 | 1,061 | 809 |
| | La3-R | AAAAAGGACACCCCAAAAGG | 6,163-6,182 | 20 | | |
| 4 | La4-F | ATTTTAGGAGGTTTAAGCCCCC | 6,142-6,163 | 22 | 674 | 674 |
| | La4-R | CATCTTACAATGCTTCAGCCCG | 6,838-6,859 | 22 | | |
| 5 | La5-F | TGGAGTTTTTATCTTGGATTGT | 6,815-6,836 | 22 | 862 | 862 |
| | La5-R | AGAAACTAGGGGCGAATTTACT | 7,699-7,720 | 22 | | |
| 6 | La6-F | ACTATTTTCTATAATCTCCCTG | 10,301-10,322 | 22 | 400 | 400 |
| | La6-R | ACAAACAGCAGACCAAAGAAAT | 10,723-10,744 | 22 | | |
| 7 | La7-F | TCCAGTGATTGAATTATATGCCT | 10,810-10,832 | 23 | 1,004 | 867 |
| | La7-R | AAATTCCAACCAAAGTCTTCCA | 11,700-11,721 | 22 | | |
| 8 | La8-F | TCCCACACCCTTATGGCAAAAT | 11,556-11,577 | 22 | 514 | 514 |
| | La8-R | AAAGATTGGCCCTGTAGAAAGT | 12,092-12,113 | 22 | | |
| 9 | La9-F | TCATGTAGGCCCTGTTTCTCTG | 12,026-12,047 | 22 | 1,312 | 819 |
| | La9-R | AGAAGGAGGGAGTATAAGGGTA | 12,867-12,888 | 22 | | |
| 10 | La10-F | TGTGGCGTAGGACTCATGAATT | 12,803-12,824 | 22 | 780 | 780 |

| | | | | | | |
|---|---|---|---|---|---|---|
| | La10-R | CCCTGCTTCTGGCATTAGGA | 13,605-13,624 | 20 | | |
| 11 | La11-F | GGTGAGCTAACGTCACCAAAAC | 13,583-13,604 | 22 | 807 | 807 |
| | La11-R | ACACCGGCAAAAATAGTCCATT | 14,412-14,433 | 22 | | |
| 12 | La12-F | GAGGATATCTTGGCAATGATCGG | 14,695-14,717 | 23 | 814 | 814 |
| | La12-R | TTCTCATTTAAACAAAGAAGGC | 15,532-15,553 | 22 | | |

*Binding site was determined based on the final mitogenome sequence.

**Table S4.** Comparison of non-coding regions of *Lingula anatina* mitogenomes from different localities.

| Locality | Non-coding regions | | | | |
|---|---|---|---|---|---|
| | **Total length of non-coding regions (bp)** | **Unassigned repeated sequences** | **Length of repeats (bp)** | **Number of Unassigned open reading frames (bp)** | **Length of ORFs** |
| Buan, Korea | 7,933 | 6 | 31-223 | 15 | 156-396 |
| Incheon, Korea | 7,934 | 6 | 31-223 | 15 | 156-396 |
| Doson, Vietnam | 8,528 | 7 | 53-223 | 17 | 153-492 |
| Yanagawa, Japan | 12,263 | 10 | 27-1,092 | 8 | 243-603 |

**Table S5.** The non-coding regions of *Lingula anatina* from Buan contains repeated sequences and open reding frames. The details of repeated sequences and open reding frames are presented in Table S7 and Table S9.

| **Non-coding region** | | **Position** | | **Size (bp)** | **Unassigned repeated sequences*** | **Unassigned open reading frame ****  |
|---|---|---|---|---|---|---|
| *cox1* | tRNA-Leu | 1,723 | 1,730 | 8 | - | - |
| tRNA-Leu | tRNA-Asp | 1,797 | 1,801 | 3 | - | - |
| tRNA-Asp | tRNA-Arg | 1,870 | 1,874 | 5 | - | - |
| tRNA-Arg | *cox2* | 1,943 | 1,943 | 1 | - | - |
| *cox2* | *nd2* | 2,694 | 2,999 | 305 | - | - |
| *nd2* | tRNA-Ala | 4,037 | 4,038 | 2 | - | - |
| tRNA-Ala | *nd4l* | 4,103 | 4,108 | 4 | - | - |
| *nd4l* | tRNA-Leu | 4,445 | 4,456 | 12 | - | - |
| tRNA-Leu | tRNA-Lys | 4,524 | 4,807 | 284 | URSB1, URSB2 | |
| tRNA-Lys | tRNA-Ser | 4,878 | 4,890 | 13 | - | - |
| tRNA-Ser | tRNA-Gln | 4,961 | 5,035 | 75 | - | - |
| tRNA-Gln | tRNA-Met | 5,103 | 6,735 | 1,633 | URSB1, URSB2, URSB2 | URFB1 to URFB4 |
| tRNA-Met | tRNA-Val | 6,808 | 6,837 | 30 | - | - |
| tRNA-Val | tRNA-Ser | 6,905 | 7,424 | 520 | - | |
| tRNA-Ser | tRNA-Thr | 7,477 | 7,624 | 148 | - | |
| tRNA-Pro | *cytb* | 9,034 | 9,106 | 73 | - | - |
| *cytb* | tRNA-Gly | 10,364 | 10,608 | 245 | URSB3 | URFB5 |
| tRNA-Gly | *atp8* | 10,673 | 10,674 | 2 | - | - |
| *atp8* | tRNA-Gly | 10,852 | 11,068 | 217 | URSB4 | - |
| tRNA-Gly | tRNA-Gly | 11,136 | 11,626 | 491 | - | URFB6 |
| tRNA-Gly | tRNA-Gly | 11,694 | 12,188 | 495 | URSB3 | URFB7 |
| tRNA-Gly | tRNA-Gly | 12,256 | 12,732 | 477 | URSB5 | URFB8 |
| tRNA-Gly | tRNA-Met | 12,800 | 13,170 | 371 | - | URFB9 |
| tRNA-Met | tRNA-Ser | 13,237 | 13,240 | 4 | - | - |
| tRNA-Ser | tRNA-Gly | 13,309 | 13,327 | 19 | - | - |

| | | | | | | |
|---|---|---|---|---|---|---|
| tRNA-Gly | tRNA-Gly | 13,396 | 14,264 | 869 | URSB6, URSB6, URSB6, URSB6, URSB6, URSB4 | URFB10 |
| tRNA-Gly | tRNA-Gly | 14,333 | 14,879 | 547 | URSB5, URSB3 | URFB11, URFB12 |
| tRNA-Gly | tRNA-Gly | 14,948 | 15,474 | 527 | URSB4 | URFB13 to URFB15 |
| tRNA-Gly | *atp6* | 15,542 | 15,546 | 5 | - | - |
| *atp6* | tRNA-Ile | 16,276 | 16,361 | 86 | - | - |
| tRNA-Ile | tRNA-Tyr | 16,433 | 16,436 | 4 | - | - |
| 16S rRNA | tRNA-Glu | 17,967 | 17,967 | 1 | - | - |
| tRNA-Glu | tRNA-Asn | 18,036 | 18,049 | 14 | - | - |
| tRNA-Asn | *nd6* | 18,120 | 18,122 | 3 | - | - |
| *nd6* | tRNA-Gly | 18,729 | 18,733 | 5 | - | - |
| tRNA-Gly | tRNA-His | 18,801 | 18,815 | 15 | - | - |
| tRNA-His | *nd1* | 18,881 | 18,930 | 50 | - | - |
| *nd1* | *nd3* | 19,840 | 19,857 | 91 | - | - |
| *nd3* | tRNA-Trp | 20,429 | 20,502 | 74 | - | - |
| tRNA_Trp | *nd5* | 20,572 | 20,581 | 10 | - | - |
| tRNA-Phe | tRNA-Trp | 22,396 | 22,411 | 16 | - | - |
| tRNA-Trp | *nd4* | 22,481 | 22,494 | 14 | - | - |
| *nd4* | *cox3* | 23,767 | 23,914 | 148 | - | - |
| *cox3* | tRNA-Cys | 24,791 | 24,802 | 12 | - | - |
| tRNA-Cys | *Cox1* | 24,871 | 14,875 | 5 | - | - |

-: Repeated sequence and open reading frame are not found.

*URSB: Unassigned repeated sequences in the Buan mitogenome; **URFB: Unassigned open reading frames in the Buan mitogenome

**Table S6.** The non-coding regions of *Lingula anatina* from Doson contains repeated sequences and open reding frames. The details of repeated sequences and open reding frames are presented in Table S8 and Table S10.

| Non-coding region | | Position | | Size (bp) | Unassigned repeated sequences * | Unassigned open reding frame** |
|---|---|---|---|---|---|---|
| *cox1* | tRNA-Leu | 1,726 | 1,730 | 5 | - | - |
| tRNA-Leu | tRNA-Asp | 1,797 | 1,801 | 3 | - | - |
| tRNA-Asp | tRNA-Arg | 1,869 | 1,871 | 3 | - | - |
| tRNA-Arg | *cox2* | 1,940 | 1,940 | 1 | - | - |
| *cox2* | *nd2* | 2,691 | 2,995 | 305 | URSD1 | - |
| *nd2* | tRNA-Ala | 4,034 | 4,943 | 910 | URSD1 | URFD1 to URFD3 |
| tRNA-Ala | *nd4l* | 5,010 | 5,012 | 3 | - | - |
| *nd4l* | tRNA-Leu | 5,349 | 5,360 | 12 | - | - |
| tRNA-Leu | tRNA-Met | 5,428 | 5,462 | 35 | - | - |
| tRNA-Met | tRNA-Gln | 5,535 | 5,541 | 7 | - | - |
| tRNA-Gln | tRNA-Met | 5,610 | 7,237 | 1,628 | URSD2, URSD2 | URFD4 to URFD6 |
| tRNA-Met | tRNA-Val | 7,310 | 7,337 | 28 | - | - |
| tRNA-Val | tRNA-Ser | 7,405 | 7,924 | 520 | - | - |
| tRNA-Ser | tRNA-Thr | 7,977 | 8,124 | 148 | - | - |
| tRNA-Pro | *cytb* | 9,533 | 9,607 | 75 | - | - |
| *cytb* | tRNA-Gly | 10,865 | 10,877 | 13 | - | - |
| tRNA-Gly | tRNA-Gly | 10,945 | 11,454 | 510 | URSD3 | URFD7 |
| tRNA-Gly | tRNA-Met | 11,522 | 11,899 | 378 | - | URFD8 |
| tRNA-Met | tRNA-Ser | 11,966 | 11,969 | 4 | - | - |
| tRNA-Ser | tRNA-Gly | 12,038 | 12,056 | 19 | - | - |
| tRNA-Gly | tRNA-Gly | 12,124 | 12,614 | 491 | URSD4 | URFD9 |

| | | | | | | |
|---|---|---|---|---|---|---|
| tRNA-Gly | tRNA-Gly | 12,683 | 14,047 | 1,365 | URSD5, URSD5, URSD5, URSD5, URSD6, URSD7, URSD3 | URFD10 to URFD12 |
| tRNA-Gly | tRNA-Gly | 14,116 | 15,470 | 1,355 | URSD4, URSD6, URSD7, URSD3 | URFD13 to URFD17 |
| tRNA-Gly | *atp8* | 15,539 | 15,542 | 4 | | - |
| *atp8* | tRNA-Gly | 15,720 | 15,964 | 245 | URSD7, URSD3 | - |
| tRNA-Gly | *atp6* | 16,033 | 16,039 | 5 | - | - |
| *atp6* | tRNA-Ile | 16,767 | 16,792 | 26 | - | - |
| tRNA-Ile | tRNA-Tyr | 16,864 | 16,867 | 4 | - | - |
| tRNA-Glu | tRNA-Asn | 18,466 | 18,479 | 14 | - | - |
| tRNA-Asn | *nd6* | 18,550 | 18,554 | 5 | - | - |
| *nd6* | tRNA-Gly | 19,159 | 19,165 | 5 | - | - |
| tRNA-Gly | tRNA-His | 19,233 | 19,249 | 17 | - | - |
| tRNA-His | *nd1* | 19,315 | 19,334 | 20 | - | - |
| *nd1* | *nd3* | 20,274 | 20,364 | 91 | - | - |
| *nd3* | tRNA-Trp | 20,863 | 20,936 | 74 | - | - |
| tRNA-Trp | *nd5* | 21,006 | 21,016 | 11 | - | - |
| tRNA-Phe | tRNA-Trp | 22,829 | 22,844 | 14 | - | - |
| tRNA-Trp | *nd4* | 22,914 | 22,927 | 14 | - | - |
| *nd4* | *cox3* | 24,200 | 24,347 | 148 | - | - |
| *cox3* | tRNA-Cys | 25,224 | 25,232 | 8 | - | - |
| tRNA-Cys | *cox1* | 25,301 | 25,305 | 5 | - | - |

-: Repeated sequence and open reading frame are not found.

*URSD: Unassigned repeated sequences in the Doson mitogenome; **URFD: unassigned open reading frames in the Doson mitogenome

**Table S7.** Unassigned repeated sequences found in non-coding regions of *Lingula anatina* from Buan, Korea.

| Unassigned repeated sequences | Length (bp) | Number of copies | A-T content (%) |
|---|---|---|---|
| URSB1 | 50 | 2 | 76 |
| URSB2 | 161, 223 | 3 | 61.4-76 |
| URSB3 | 86, 158 | 2 | 66.3-71.5 |
| URSB4 | 131 | 3 | 69-72.5 |
| URSB5 | 31 | 2 | 67.7 |
| URSB6 | 75 | 5 | 40-41.3 |

**Table S8.** Unassigned repeated sequences found in non-coding regions of *Lingula anatina* from Doson, Vietnam.

| Unassigned repeated sequences | Length (bp) | Number of copies | A-T content (%) |
|---|---|---|---|
| URSD1 | 80, 83 | 2 | 82.5-83.1 |
| URSD2 | 223 | 2 | 61.9 |
| URSD3 | 76,77 | 4 | 64.9-65.8 |
| URSD4 | 85 | 2 | 62.4-69.4 |
| URSD5 | 53,75 | 4 | 41.5-46.7 |
| URSD6 | 102, 104 | 2 | 74.5-75 |
| URSD7 | 164 | 3 | 70.7-72 |

**Table S9.** Unassigned open reading frames (≥50 aa) found in non-coding regions of *Lingula anatina* from Buan, Korea.

| Unassigned open reading frames | Start | Stop | Nucleotide length | Amino acid length | Strand |
|---|---|---|---|---|---|
| URFB1 | 5,177 | 5,527 | 381 | 126 | - |
| URFB2 | 6,277 | 6,432 | 156 | 51 | - |
| URFB3 | 6,367 | 6,618 | 252 | 83 | + |
| URFB4 | 6,500 | 6,655 | 156 | 51 | - |
| URFB5 | 10,433 | 10,606 | 174 | 57 | + |
| URFB6 | 11,141 | 11,506 | 366 | 121 | + |
| URFB7 | 11,699 | 12,061 | 363 | 120 | + |
| URFB8 | 12,261 | 12,590 | 330 | 109 | + |
| URFB9 | 12,802 | 13,047 | 246 | 81 | + |
| URFB10 | 13,610 | 14,005 | 396 | 131 | + |
| URFB11 | 14,334 | 14,660 | 327 | 108 | + |
| URFB12 | 14,569 | 14,877 | 309 | 102 | + |
| URFB13 | 14,949 | 15,278 | 330 | 109 | + |
| URFB14 | 15,008 | 15,289 | 282 | 93 | - |
| URFB15 | 15,094 | 15,291 | 198 | 62 | + |

**Table S10.** Unassigned open reading frames (≥50 aa) found in non-coding regions of *Lingula anatina* from Doson, Vietnam.

| **Unassigned open reading frames** | **Start** | **Stop** | **Nucleotide length** | **Amino acid length** | **Strand** |
|---|---|---|---|---|---|
| URFD1 | 4,233 | 4,715 | 483 | 160 | + |
| URFD2 | 4,458 | 4,631 | 174 | 57 | - |
| URFD3 | 4,489 | 4,722 | 234 | 77 | + |
| URFD4 | 5,762 | 5,914 | 153 | 50 | + |
| URFD5 | 5,816 | 6,064 | 249 | 85 | - |
| URFD6 | 6,784 | 6,939 | 156 | 51 | - |
| URFD7 | 10,949 | 11,311 | 363 | 120 | + |
| URFD8 | 11,526 | 11,768 | 243 | 80 | + |
| URFD9 | 12,129 | 12,494 | 366 | 121 | + |
| URFD10 | 12,684 | 13,175 | 492 | 163 | + |
| URFD11 | 13,230 | 13,550 | 321 | 106 | - |
| URFD12 | 13,591 | 13,920 | 330 | 109 | + |
| URFD13 | 14,117 | 14,449 | 333 | 110 | + |
| URFD14 | 14,176 | 14,466 | 291 | 96 | - |
| URFD15 | 14,283 | 14,525 | 243 | 80 | + |
| URFD16 | 14,690 | 15,016 | 327 | 108 | + |
| URFD17 | 14,925 | 15,356 | 432 | 143 | + |
